# Association of ICD-10-Coded Pneumonia Events with Interstitial Lung Disease Outcomes in Patients with Rheumatoid Arthritis: A Large Database Retrospective Cohort

**DOI:** 10.3390/arm94040049

**Published:** 2026-07-22

**Authors:** Esteban Kosak Lopez, Luis Rodriguez Donís, Justin Lam, Andrew Geller, Raul Leguizamon, Michael Vera Ricaurte, Priscilla Nethala, Maria Planchart Ferretto, Maria Laura Fernandez-Wever, Jose M. Martinez-Manzano, Enrique Pacheco, Shahrzad Abdollahi

**Affiliations:** 1 Medicine Department, Jefferson Einstein Philadelphia Hospital, Philadelphia, PA 19141, USA; justin.lam@jefferson.edu (J.L.); andrew.geller@jefferson.edu (A.G.); raul.leguizamon@jefferson.edu (R.L.); michaeldejesus.veraricaurte@jefferson.edu (M.V.R.); priscilla.nethala@jefferson.edu (P.N.); maria.planchartferretto@jefferson.edu (M.P.F.); 2 Instituto de Biomedicina, Universidad Central de Venezuela, Caracas 1051, Venezuela; lurarodo@gmail.com; 3 Pulmonary and Critical Care Division, Brigham and Women’s Hospital, Boston, MA 02115, USA; mfernandezwever@bwh.harvard.edu (M.L.F.-W.); jmartinezmanzano@bwh.harvard.edu (J.M.M.-M.); 4 Pulmonary and Critical Care Division, Jefferson Einstein Philadelphia Hospital, Philadelphia, PA 19141, USA; enrique.pacheco@jefferson.edu; 5 Rheumatology Division, Jefferson Einstein Philadelphia Hospital, Philadelphia, PA 19141, USA; shahrzad.abdollahi@jefferson.edu

**Keywords:** rheumatoid arthritis, pneumonia, interstitial lung disease, prevention

## Abstract

**Highlights:**

**What are the main findings?**

- Patients with rheumatoid arthritis who have an ICD-10-coded pneumonia event (CPE) within one year of diagnosis have a significantly increased risk of a subsequent diagnosis of interstitial lung disease (HR = 2.87) and pulmonary fibrosis (HR = 2.48).
- A CPE in this patient population is also linked to a nearly two-fold higher risk of all-cause mortality, alongside increased incidences of acute and chronic hypoxic respiratory failure.

**What are the implications of the main findings?**

- Because a CPE during early rheumatoid arthritis may represent the first clinical recognition of pre-existing or emerging interstitial lung disease rather than a true infectious episode, it serves as a critical marker warranting pulmonary surveillance.
- While preventative measures like vaccinations remain important, a CPE should primarily serve as a clinical warning sign to prompt a more thorough pulmonary examination for latent interstitial lung disease.

**Abstract:**

**Introduction:** Patients with rheumatoid arthritis (RA) have higher risk for pneumonia, interstitial lung disease (ILD) and pulmonary fibrosis (PF). However, the association between an ICD-10-coded pneumonia event (CPE) and the incidence of ILD or PF in the RA population remains unclear. **Methods:** We conducted a retrospective cohort study using the TriNetX database. Patients with ICD-10 for RA aged 50 or older who had a CPE within one year of RA diagnosis (CPE cohort, n = 4553) were matched 1:1 by propensity score for key factors, including demographics, comorbidities (i.e., COPD), and medication use (DMARDs, corticosteroids) to RA patients without a CPE (Control cohort, n = 4553). Cox proportional hazard models assessed the incidence of a composite ILD outcome, PF, and secondary complications over a 4-year follow-up after the index event defined as 1-year after RA diagnosis for both cohorts. **Results:** The CPE cohort showed an increased risk for all outcomes. Patients with CPE had a 2.48-fold increased risk for PF (HR = 2.48; 95% CI, 1.78–3.45; *p* < 0.01) and a 2.87-fold increased risk for the composite ILD outcome (HR = 2.87; 95% CI, 2.18–3.80; *p* < 0.01). The risk of rheumatoid lung disease was 4.15 times higher (HR = 4.15; 95% CI, 2.30–7.50; *p* < 0.01). Furthermore, the CPE group had a higher risk for all-cause mortality (HR = 1.82; 95% CI, 1.58–2.09; *p* < 0.01). **Conclusions:** The CPE within one year of RA diagnosis is associated with an increase in subsequent ILD-coded outcomes. While this retrospective design cannot establish causality, an unspecified pneumonia code in early RA may represent an early clinical manifestation of unrecognized ILD, serving as a high-risk marker that warrants pulmonary surveillance.

## 1. Introduction

Interstitial lung disease (ILD) is a well-recognized complication associated with adverse outcomes and increased mortality in patients with rheumatoid arthritis (RA) [1,2]. Concurrently, patients with RA have an elevated risk for pulmonary infections [3], which account for a substantial proportion of RA-related hospital admissions [4]. While previous research in the general population has demonstrated that a history of pneumonia is independently associated with an increased risk of developing ILD [5], less is known about the clinical significance of prior pneumonia episodes and their temporal association with the subsequent development of ILD or pulmonary fibrosis (PF) in the RA population.

When investigating this relationship using administrative healthcare databases, it is critical to distinguish between a true infectious pneumonia acting as a biological or pathological antecedent to fibrosis, versus a coded pneumonia event (CPE) serving as an associative marker. Such a diagnostic profile—an unspecified pneumonia code in early RA—may act as a surrogate marker of high disease activity, immunosuppression, frailty, and unrecognized cardiopulmonary comorbidity [6,7,8]. Furthermore, identifying these events is clinically relevant, as a CPE may not only reflect an infection but could also represent an early, unrecognized clinical manifestation of emerging RA-ILD [9].

To our knowledge, the specific link between early CPE and subsequent ILD has not been described in patients with RA. Hereby, we aim to determine in a large retrospective cohort whether an episode of CPE within the first year of RA diagnosis increases the risk for subsequent ILD and PF outcomes. We sought to investigate whether these patients have different pulmonary and survival trajectories compared to those without a CPE.

## 2. Material and Methods

This retrospective cohort study used data from the TriNetX database (TriNetX LLC, Cambridge, MA, USA), which provides access to de-identified inpatient and outpatient electronic medical records from healthcare organizations worldwide. The database aggregates inpatient and outpatient encounters, diagnoses, medications, and laboratory results with a waiver from the Western Institutional Review Board for real-time data of electronic medical records. This analysis was conducted on the TriNetX USA Collaborative Network, consisting of 70 health care organizations, and was collected in October 2025. Further details on the network have been described elsewhere [10].

Patients aged 50 years or older with a diagnosis of RA [International Statistical Classification of Diseases, Tenth Revision (ICD10) code M05 or M06] who had inpatient or outpatient healthcare center visits were included. Patients were divided into two cohorts based on episodes of CPE with unspecified organisms (ICD10CM:J18). CPE cohort had a CPE within one year after diagnosis of RA. Conversely, control cohort did not have a CPE one year after RA diagnosis. Both cohorts required the use of any of the current RA treatments, such as conventional synthetic disease-modifying antirheumatic drugs (DMARDs), including methotrexate, sulfasalazine, leflunomide, hydroxychloroquine; biologic DMARDs, including adalimumab, certolizumab pegol, etanercept, golimumab, infliximab, tocilizumab, sarilumab, anakinra, abatacept, and rituximab; targeted synthetic DMARDs, including baricitinib, tofacitinib, upadacitinib. This was done to ensure a more accurate RA diagnosis.

The characteristics for the cohorts were obtained up to 1 year before an RA diagnosis, including the day of diagnosis. These cohorts were compared by using 1:1 propensity score matching on demographic characteristics [age at index, sex, race, body mass index (BMI)], comorbidities (COPD, cardiovascular comorbidities, history of nicotine dependence), medications (corticosteroids, conventional synthetic, biological, and targeted synthetic DMARDs), and laboratory values [blood rheumatoid factor titer, blood CCP antibody titer, C-reactive protein titer (CRP), erythrocyte sedimentation rate (ESR)] to minimize confounding (Table 1).

The clinical outcomes of interest were diagnosis ILD as a composite outcome [other specified interstitial pulmonary diseases (ICD10CM:J84.8), interstitial pulmonary disease, unspecified (ICD10CM:J84.9), rheumatoid lung disease with rheumatoid arthritis (ICD10CM:M05.1), and idiopathic non-specific interstitial pneumonitis (ICD10CM:J84.113)], and PF diagnosis [pulmonary fibrosis, unspecified (ICD10CM:J84.10), other interstitial pulmonary diseases with fibrosis (ICD10CM:J84.1), interstitial lung disease with progressive fibrotic phenotype in diseases classified elsewhere (ICD10CM:J84.170), other interstitial pulmonary diseases with fibrosis in diseases classified elsewhere (ICD10CM:J84.178)]. Secondary outcomes included mortality (captured as “deceased” in TriNetX platform), acute hypoxic respiratory failure (AHRF) (ICD10CM:J96.01), chronic hypoxic respiratory failure (CHRF) (ICD10CM:J96.11), pulmonary hypertension incidence (ICD10CM:I27.20), and rheumatoid lung disease (RLD) (ICD10CM:M05.1) as an important separate outcome. Because our exposure and outcomes of interest were defined exclusively using administrative ICD-10 codes without radiographic or microbiological confirmation, there is an inherent risk of misclassification bias in the cohort allocation and outcome ascertainment.

**Figure 1:**
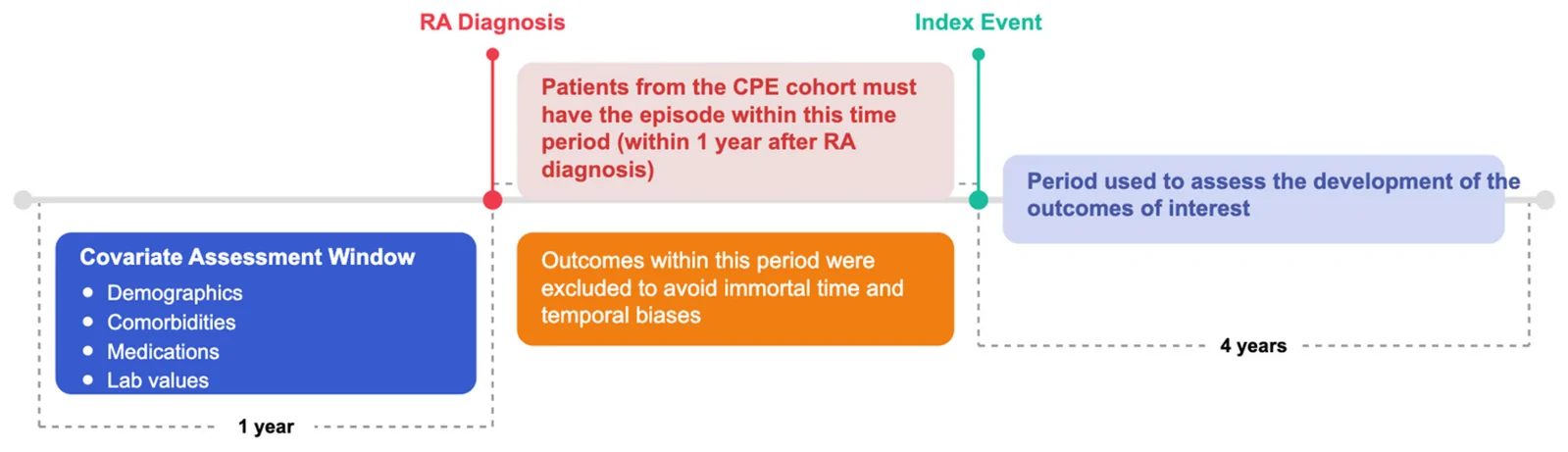
Graphical depiction of study design; Abbreviations: rheumatoid arthritis (RA), coded pneumonia event (CPE).

The index event for each cohort was defined as outcomes 1 year after RA diagnosis. Patients with outcomes prior to the index event were excluded from the analysis (Table 2). This was done to avoid immortal time and temporal biases. Only events occurring within the prior 20 years were considered. From the moment of the index event, a time window of 1460 days (4 years) was used to assess the development of the outcomes of interest. A clear definition of cohort creation and index event can be seen in Figure 1.

**Figure 2:**
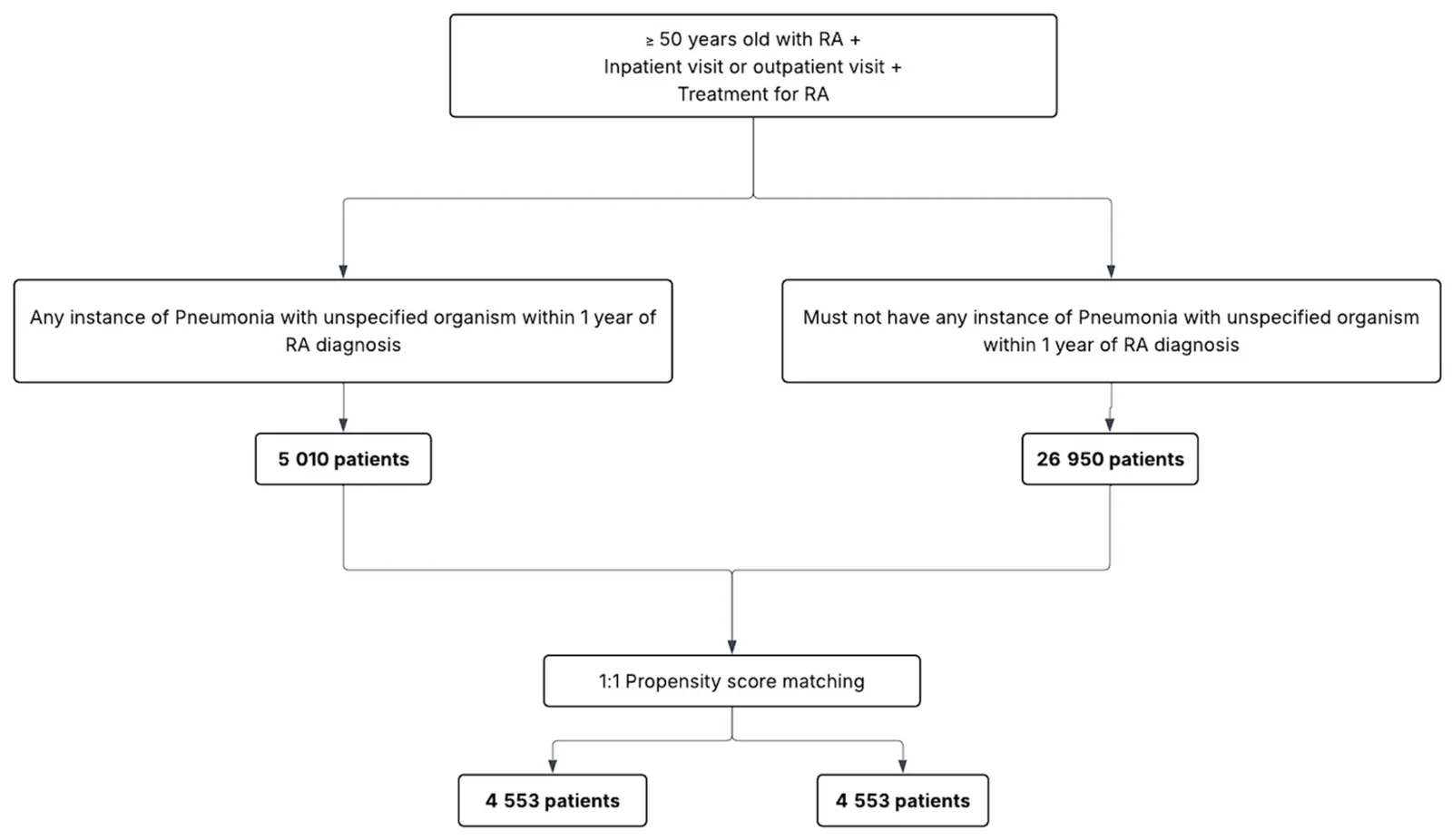
Patient selection process; Abbreviations: rheumatoid arthritis (RA).

**Figure 3:**
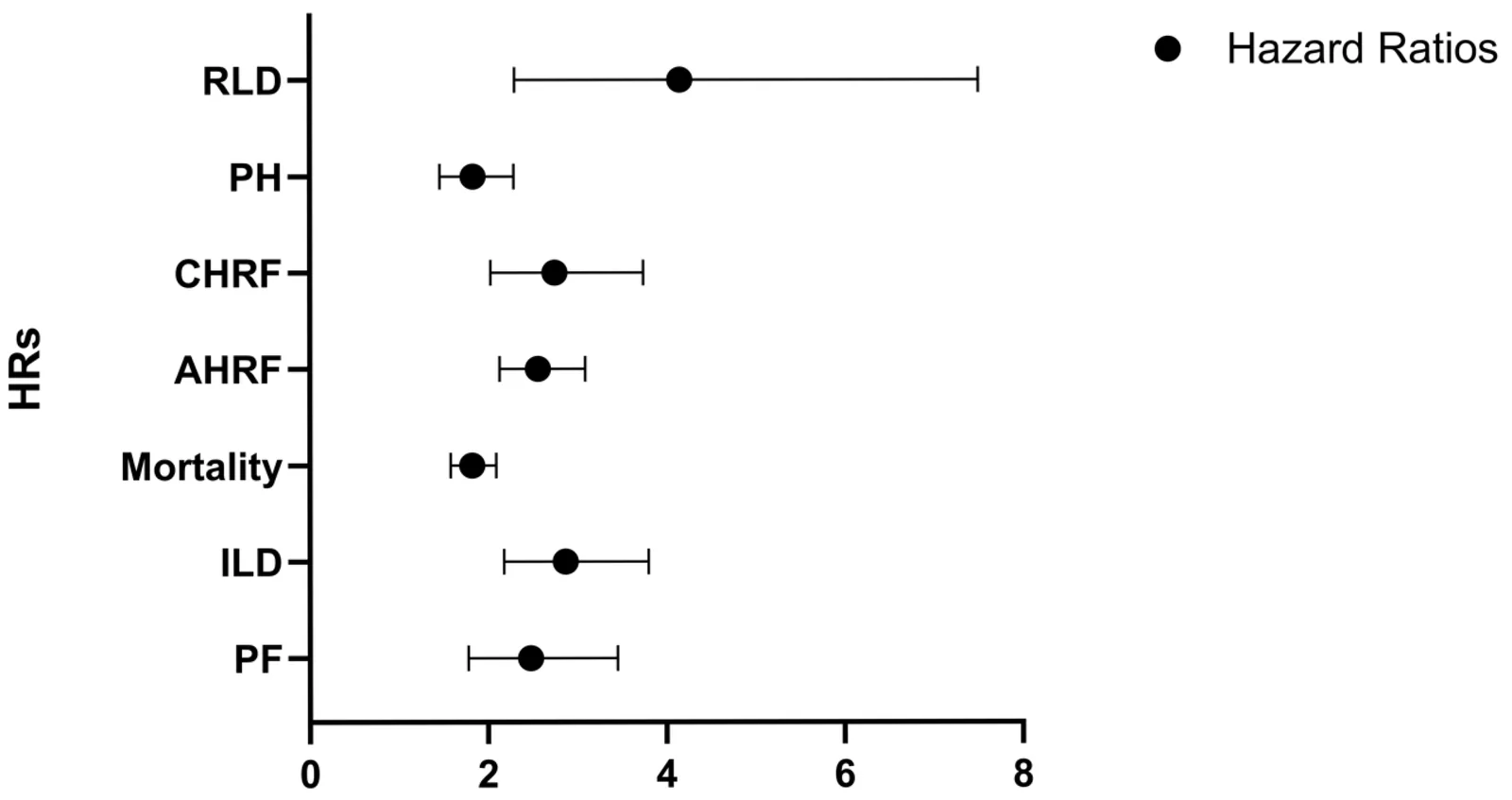
HRs of primary and secondary outcomes. AHRF, acute hypoxic respiratory failure; CHRF, chronic hypoxic respiratory failure; PH, pulmonary hypertension; RLD, rheumatoid lung disease.

Baseline characteristics for all cohorts were reported using means and standard deviations (SDs) for continuous variables and counts and percentages for categorical variables. The covariate balance between groups was assessed using the standardized mean difference (SMD); an SMD < 0.1 was considered indicative of a good match. Cox proportional hazard models were used to compare the incidence of each outcome between the CPE and control groups, producing hazard ratios (HRs) with 95% confidence intervals (CIs). A two-sided *p*-value of <0.05 was considered statistically significant. All analyses were done using the statistical tools within the TriNetX platform, including propensity score matching. The platform utilizes logistic regression to calculate propensity scores based on the selected covariates and employs a 1:1 nearest-neighbor greedy matching algorithm. The model utilized main effects without interaction terms, and cluster-robust standard errors were not applied. Graphical depictions of the study design and patient selection process (Figure 1 and Figure 2) were created using Figma (Figma, Inc., San Francisco, CA, USA). Data visualization for clinical outcomes (Figure 3) was performed using GraphPad Prism version 11.0.2 (GraphPad Software, Boston, MA, USA).

Due to the nature of the TriNetX database, not all information is uniformly available for every patient, particularly for certain laboratory values. Therefore, the patient characteristics and laboratory values used for propensity score matching and presented in the results were restricted to the data returned by the platform.

## 3. Results

The characteristics of the cohort before propensity score matching is in Table 1. After inclusion and exclusion criteria were applied to the TriNetx database, 1:1 propensity score matching was performed, resulting in two cohorts for analysis: CPE cohort (4553 patients with RA and a CPE episode within 1 year of RA diagnosis) and control cohort (4553 patients with RA and no CPE episode within 1 year of RA diagnosis). The patient selection process is detailed in Figure 2.

The baseline demographic and clinical characteristics of the matched cohorts are shown in Table 1. The patient population had an average index age of 66.2 years, was mostly female (about 68%), and white (about 78%). Common comorbidities included essential hypertension (around 66%), hyperlipidemia (roughly 45%), a history of nicotine dependence (about 29%), type 2 diabetes mellitus (approximately 28%), chronic ischemic heart disease (about 26%), and chronic obstructive pulmonary disease (roughly 20%) for CPE and control, respectively.

The most commonly prescribed medications were prednisone (about 49%), methylprednisolone (around 34%), methotrexate (approximately 29%), hydroxychloroquine (about 24%), and triamcinolone (about 17%). Concerning laboratory values, the average rheumatoid factor (RF) was approximately 30.8 IU/mL, the average cyclic citrullinated peptide was about 50 U/mL, and the average erythrocyte sedimentation rate was around 31 mm/hr. While most covariates achieved excellent balance (SMD < 0.1), a slight residual imbalance persisted for C-reactive protein (SMD = 0.21) with an average of 35.9 vs. 24.4 mg/L for CPE and control, respectively.

Over the 4-year follow-up period, the incidence of both ILD and PF was higher in the CPE cohort compared to its matched control. To accurately assess incident risk, patients with a recorded diagnosis of a specific outcome prior to the index event were excluded from the denominator for that specific analysis (Table 2). For the outcomes of interest, the number of cases (CPE vs. control) was 110/3945 (2.8%) vs. 52/4278 (1.2%) for PF, 166/3846 (4.3%) vs. 71/4319 (1.6%) for composite ILD, 527/3902 (13.5%) vs. 323/4326 (7.5%) for mortality, 321/3158 (10.2%) vs. 171/4178 (4.1%) for AHRF, 141/4271 (3.3%) vs. 58/4465 (1.3%) for CHRF, 198/4070 (4.9%) vs. 124/4293 (2.9%) for pulmonary hypertension, and 52/4371 (1.18%) vs 14/4523 (0.3%) for rheumatoid lung disease.

For the CPE group, the HR for the clinical outcomes of interest were as follows: PF, HR = 2.48 (95% CI, 1.78–3.45; *p* < 0.01); ILD, HR = 2.87 (95% CI, 2.18–3.80; *p* < 0.01); mortality, HR = 1.82 (95% CI, 1.58–2.09; *p* < 0.01); AHRF, HR = 2.56 (95% CI, 2.13–3.09; *p* < 0.01); CHRF, HR = 2.75 (95% CI, 2.03–3.74; *p* < 0.01); pulmonary hypertension, HR = 1.83 (95% CI, 1.46–2.29; *p* < 0.01), and RLD, HR = 4.15 (95% CI, 2.30–7.50; *p* < 0.01). These results are presented as forest plots in Figure 3.

## 4. Discussion

Our retrospective cohort study investigated the association between a CPE within one year after RA diagnosis and the subsequent risk of ILD and PF development in patients with RA. We observed a 2.48-fold increased risk for PF, a 2.87-fold increased risk for ILD, and a 4.15-fold increased risk for rheumatoid lung disease. Furthermore, our analysis revealed that a CPE was associated with a nearly three-fold increase in all-cause mortality and an elevated risk for other serious pulmonary complications, including acute and chronic respiratory failure and pulmonary hypertension.

Pneumonia is a known independent risk factor for mortality in patients with RA and increases the risk for polymicrobial infections as well in this patient population [11]. These findings are consistent with evidence showing associations between prior respiratory events and the incidence of ILD [5]. Previous studies have established a history of CPE as a risk factor for the incidence of ILD in the general population, and more broadly, pulmonary comorbidities have been identified as significant risk factors for ILD in patients with RA [5,12]. However, to the best of our knowledge, no prior research has specifically investigated the association between a CPE and the subsequent development of ILD or PF in the RA population. Our study, therefore, tried to address this gap by leveraging a large dataset.

However, it is crucial to recognize that the use of ICD-10 codes for pneumonia (specifically the J18.x series) in large administrative datasets often serves as a surrogate for an acute respiratory syndrome rather than a microbiologically confirmed infection [13]. In clinical practice, pneumonia is primarily a clinical diagnosis based on symptoms and radiographic opacities, which overlap significantly with other pulmonary processes common in RA. Specifically, early manifestations of RA-ILD, such as organizing pneumonia, acute exacerbations of subclinical fibrosis, or non-specific interstitial pneumonia, can pose as infectious pneumonia. These conditions are frequently misdiagnosed as pneumonia for many months before the underlying interstitial or fibrotic process is correctly identified [14]. Therefore, the CPE captured in this study may, in many instances, represent the first clinical recognition of pre-existing or emerging ILD rather than a distinct antecedent infectious episode. Overall, the high hazard ratios and baseline pulmonary imbalances suggest that many CPE in our study may actually represent initial manifestations of ILD rather than true infectious pneumonia. This risk of reverse causation is a major challenge in administrative dataset research for RA patients.

Regarding the incidence of ILD and PF in our cohort (4.3% and 2.8% over 3 years, respectively), it is lower than the pooled prevalence estimates from meta-analyses, which range from 15 to 21% for RA-ILD, but aligns with real-world incidence rates reported in large administrative and clinical datasets [1,12,15,16,17]. Several factors could explain this; for instance, our population was older (mean age 71), predominantly female, and had high rates of comorbidities and DMARD/corticosteroid use. Older age and male sex are risk factors for RA-ILD, but female predominance may lower overall incidence [12]. Most meta-analyses report cumulative prevalence over much longer periods (10–20 years), while our study assessed incident cases over 4 years after the index event (total 5 years from RA diagnosis), likely lowering the incidence [17]. Finally, high CRP/ESR and seropositivity (RF, anti-CCP) are risk factors for ILD, but incomplete data on these markers in our cohort may limit risk stratification [12].

The baseline data revealed a substantial number of patients with pre-existing ILD, PF, and AHRF, with these conditions being more common in the pneumonia cohort (Table 2). This aligns with a previous study reporting that up to 13.5% of RA patients are diagnosed with ILD before their RA diagnosis [18]. The prevalence of corticosteroid use was also high across both cohorts, raising concerns about uncontrolled disease activity, particularly among those with CPE. Despite this, the low overall prevalence of CHRF suggests that the cohorts, while having significant comorbidities, may not represent the most severely ill patients with advanced lung disease, which could influence the generalizability of the findings.

Furthermore, a major methodological limitation in this study is the inability to directly adjust for dynamic RA disease activity, which is an independent risk factor for RA-ILD. While our propensity score matching accounted for established risk factors like seropositivity and prior medication use, it could not track precise, longitudinal inflammatory markers. Specifically, our data indicate that both cohorts exhibited high baseline inflammation, with mean CRP values exceeding the commonly cited threshold of 10 mg/L (typically indicative of high disease activity) [19]. Therefore, while we cannot definitively rule out residual confounding by persistently high RA disease activity, our study suggests that a CPE could be a clinical marker for elevated ILD risk.

Our study had several limitations. First, it was not possible to confirm the precision of any electronic health records in the TriNetX platform. The diagnoses and the clinical outcomes were based on the ICD-10 coding system; therefore, these are prone to misclassification bias as they rely on the accuracy of the physician’s documentation. Second, we tracked outcomes from a fixed index event and did not assess the severity of the initial episode or account for the occurrence of subsequent, recurrent CPE after the first year, which could independently impact disease progression. Third, there is a significant risk of surveillance and detection bias; patients presenting with a CPE are more likely to undergo subsequent chest imaging and pulmonology follow-up, naturally leading to a higher detection rate of subclinical ILD compared to the control group. Fourth, mortality acts as a significant competing risk in this population. Because analyses were restricted to the native tools within the TriNetX platform, we utilized standard Cox proportional hazard models and were unable to perform Fine-Gray subdistribution hazard models to adjust for nonfatal outcome detection. Fifth, TrinetX has a major limitation in that not all information for patients is available.

Furthermore, the lack of granular diagnostic data, such as high-resolution computed tomography scans or pulmonary function testing, which are the clinical gold standards for diagnosing ILD. Our outcomes rely on a broad cluster of ICD-10 codes, which may aggregate distinct clinical entities such as organizing pneumonia, NSIP, or acute inflammatory lung injury. Without radiographic or histopathologic confirmation, it is impossible to distinguish between these heterogeneous processes or determine if the coded outcomes represent true chronic fibrotic disease versus transient post-infectious parenchymal changes. Lastly, due to the study’s retrospective nature, our findings only describe associations between CPE and outcomes but not causation.

Our findings suggest that a history of CPE should be considered a warning sign in RA patients for possible underlying ILD, justifying more frequent screening, potentially facilitating timely diagnosis and intervention.

## 5. Interpretation

In this large-scale analysis of electronic health records, coded pneumonia event (CPE) within one year of rheumatoid arthritis (RA) diagnosis was associated with a higher risk of subsequent ILD and pulmonary fibrosis-coded outcomes. However, the high hazard ratios and baseline pulmonary imbalances suggest that these CPE may represent early clinical manifestations or misdiagnoses of emerging RA-ILD rather than independent infectious triggers. Given the difficulty in distinguishing acute infection from early-stage interstitial lung disease in RA patients based on administrative data alone, these findings highlight a ‘red flag’ for clinicians. A history of pneumonia in early RA should prompt pulmonary screening to facilitate timely diagnosis of potentially underlying interstitial lung disease.

## Figures and Tables

**Table 1:** Cohort characteristics before and after propensity-score matching.

Variable	Before PSM			After PSM		
	**RA + CPE Cohort** **(n = 5010)**	**RA + No CPE Cohort** **(n = 26,950)**	**SMD**	**RA + CPE Cohort** **(n = 4553)**	**RA + No CPE Cohort** **(n = 4553)**	**SMD**
Age at index, mean ± SD	66.8 ± 10.7	60.5 ± 10.2	0.602	66.2 ± 10.6	66.2 ± 10.6	0.006
**Demographics, n (%)**						
Female	3415 (68.2%)	19,437 (72.1%)	0.087	3107 (68.2%)	3082 (67.7%)	0.012
Male	1595 (31.8%)	7513 (27.9%)	0.087	1446 (31.8%)	1471 (32.3%)	0.012
White	3915 (78.1%)	20,731 (76.9%)	0.029	3555 (78.1%)	3557 (78.1%)	0.001
Black or African American	647 (12.9%)	3161 (11.7%)	0.036	578 (12.7%)	564 (12.4%)	0.009
Hispanic or Latino	181 (3.6%)	1507 (5.6%)	0.095	176 (3.9%)	172 (3.8%)	0.005
Not Hispanic or Latino	4288 (85.6%)	22,114 (82.1%)	0.096	3877 (85.2%)	3819 (83.9%)	0.035
**Diagnosis, n (%)**						
Essential hypertension	3456 (69.0%)	12,081 (44.8%)	0.503	3043 (66.8%)	3042 (66.8%)	<0.001
Chronic ischemic heart disease	1488 (29.7%)	2978 (11.1%)	0.476	1217 (26.7%)	1188 (26.1%)	0.014
Heart failure, unspecified	790 (15.8%)	934 (3.5%)	0.427	560 (12.3%)	533 (11.7%)	0.018
Cerebral infarction	286 (5.7%)	509 (1.9%)	0.201	242 (5.3%)	187 (4.1%)	0.057
Hyperlipidemia, unspecified	2413 (48.2%)	7307 (27.1%)	0.445	2075 (45.6%)	2052 (45.1%)	0.010
Type 2 diabetes mellitus	1516 (30.3%)	4944 (18.3%)	0.281	1309 (28.8%)	1313 (28.8%)	0.002
Asthma	1075 (21.5%)	2870 (10.6%)	0.298	955 (21.0%)	725 (15.9%)	0.131
Chronic obstructive pulmonary disease, unspecified	1259 (25.1%)	1628 (6.0%)	0.546	952 (20.9%)	921 (20.2%)	0.017
Personal history of nicotine dependence	1662 (33.2%)	3193 (11.8%)	0.528	1350 (29.7%)	1296 (28.5%)	0.026
**Medication**						
Prednisone	2572 (51.3%)	8863 (32.9%)	0.38	2244 (49.3%)	2240 (49.2%)	0.002
Methylprednisolone	1861 (37.1%)	4795 (17.8%)	0.444	1570 (34.5%)	1581 (34.7%)	0.005
Dexamethasone	1309 (26.1%)	3374 (12.5%)	0.35	1113 (24.4%)	1134 (24.9%)	0.011
Budesonide	669 (13.4%)	928 (3.4%)	0.363	495 (10.9%)	463 (10.2%)	0.023
Hydrocortisone	684 (13.7%)	1298 (4.8%)	0.309	541 (11.9%)	520 (11.4%)	0.014
Triamcinolone	893 (17.8%)	3274 (12.1%)	0.16	794 (17.4%)	813 (17.9%)	0.011
Prednisolone	177 (3.5%)	449 (1.7%)	0.118	156 (3.4%)	174 (3.8%)	0.021
Betamethasone	277 (5.5%)	1166 (4.3%)	0.056	248 (5.4%)	252 (5.5%)	0.004
Tacrolimus	74 (1.5%)	181 (0.7%)	0.078	66 (1.4%)	42 (0.9%)	0.049
Cyclosporine	74 (1.5%)	237 (0.9%)	0.055	65 (1.4%)	51 (1.1%)	0.027
Azathioprine	90 (1.8%)	239 (0.9%)	0.079	80 (1.8%)	47 (1.0%)	0.062
Mycophenolate mofetil	67 (1.3%)	149 (0.6%)	0.081	61 (1.3%)	46 (1.0%)	0.031
Methotrexate	1486 (29.7%)	6324 (23.5%)	0.141	1317 (28.9%)	1359 (29.8%)	0.020
Etanercept	239 (4.8%)	1034 (3.8%)	0.046	221 (4.9%)	110 (2.4%)	0.131
Adalimumab	335 (6.7%)	1272 (4.7%)	0.085	314 (6.9%)	209 (4.6%)	0.099
Golimumab	59 (1.2%)	128 (0.5%)	0.078	50 (1.1%)	22 (0.5%)	0.069
Anakinra	13 (0.3%)	34 (0.1%)	0.030	10 (0.2%)	14 (0.3%)	0.017
Certolizumab	10 (0.2%)	10 (0.0%)	0.047	10 (0.2%)	10 (0.2%)	<0.001
Infliximab	135 (2.7%)	408 (1.5%)	0.082	119 (2.6%)	75 (1.6%)	0.067
Leflunomide	475 (9.5%)	1347 (5.0%)	0.174	412 (9.0%)	279 (6.1%)	0.110
Sulfasalazine	373 (7.4%)	1546 (5.7%)	0.069	333 (7.3%)	272 (6.0%)	0.054
Hydroxychloroquine	1237 (24.7%)	5739 (21.3%)	0.081	1111 (24.4%)	1089 (23.9%)	0.011
Certolizumab pegol	48 (1.0%)	127 (0.5%)	0.058	47 (1.0%)	19 (0.4%)	0.073
Abatacept	169 (3.4%)	339 (1.3%)	0.141	151 (3.3%)	55 (1.2%)	0.142
Rituximab	172 (3.4%)	194 (0.7%)	0.191	155 (3.4%)	51 (1.1%)	0.154
Baricitinib	20 (0.4%)	10 (0.0%)	0.078	15 (0.3%)	10 (0.2%)	0.021
Tofacitinib	161 (3.2%)	286 (1.1%)	0.149	150 (3.3%)	32 (0.7%)	0.186
Upadacitinib	51 (1.0%)	66 (0.2%)	0.098	45 (1.0%)	22 (0.5%)	0.059
**Laboratory, mean ± SD**						
BMI	29.8 ± 8.0 (77.8%)	31.0 ± 7.5 (73.8%)	0.157	29.9 ± 8.0 (78.3%)	31.1 ± 7.6 (79.2%)	0.155
Rheumatoid factor (Units/volume) in serum or plasma	30.1 ± 24.4 (3.6%)	29.8 ± 25.5 (8.1%)	0.013	30.6 ± 24.9 (3.7%)	31.1 ± 25.4 (9.1%)	0.020
Cyclic citrullinated peptide Ab.IgG (Units/volume) in serum, plasma, or blood	54.4 ± 76.1 (2.2%)	42.6 ± 69.0 (4.6%)	0.163	49.4 ± 69.6 (2.4%)	51.3 ± 78.9 (1.9%)	0.025
C-reactive protein (Mass/volume) in serum, plasma, or blood	37.1 ± 62.2 (46.2%)	18.6 ± 36.7 (37.2%)	0.362	35.9 ± 62.1 (45%)	24.4 ± 45.2 (46%)	0.210
Erythrocyte sedimentation rate	32.4 ± 28.1 (46.8%)	26.3 ± 24.9 (42.6%)	0.233	31.5 ± 27.5 (46.4%)	30.6 ± 26.3 (47%)	0.032

**Table 2:** Analytical cohort sizes and incident events following exclusion of patients with prior disease.

Clinical Outcome	Cohort	Initial N	Excluded (Event Prior To Index)
Pulmonary fibrosis	Coded pneumonia	4553	608 (13.35%)
	Control	4553	275 (6.03%)
Interstitial lung disease	Coded pneumonia	4553	707 (15.52%)
	Control	4553	234 (5.13%)
Mortality	Coded pneumonia	4553	651 (14.29%)
	Control	4553	227 (4.98%)
Acute hypoxic respiratory failure	Coded pneumonia	4553	1395 (30.6%)
	Control	4553	375 (8.2%)
Chronic hypoxic respiratory failure	Coded pneumonia	4553	282 (6.19%)
	Control	4553	88 (1.9%)
Pulmonary hypertension	Coded pneumonia	4553	483 (10.6%)
	Control	4553	260 (5.71%)
Rheumatoid lung disease	Coded pneumonia	4553	182 (3.99%)
	Control	4553	30 (0.65%)

## Data Availability

The datasets presented in this article are not readily available because of third party commercial restrictions by TrinetX. Requests to access the datasets should be directed to www.trinetx.com.
